# From Pixels to Prognosis: A Narrative Review on Artificial Intelligence’s Pioneering Role in Colorectal Carcinoma Histopathology

**DOI:** 10.7759/cureus.59171

**Published:** 2024-04-27

**Authors:** Suhit Naseri, Samarth Shukla, KM Hiwale, Miheer M Jagtap, Pravin Gadkari, Kartik Gupta, Mamta Deshmukh, Shakti Sagar

**Affiliations:** 1 Pathology, Jawaharlal Nehru Medical College, Datta Meghe Institute of Higher Education & Research, Wardha, IND; 2 Radiation Oncology, Delhi State Cancer Institute, Delhi, IND; 3 Pathology, Indian Institute of Medical Sciences and Research, Jalna, IND

**Keywords:** image analysis, prognosis, diagnosis, artificial intelligence (ai), histopathology, colorectal carcinoma

## Abstract

Colorectal carcinoma, a prevalent and deadly malignancy, necessitates precise histopathological assessment for effective diagnosis and prognosis. Artificial intelligence (AI) emerges as a transformative force in this realm, offering innovative solutions to enhance traditional histopathological methods. This narrative review explores AI's pioneering role in colorectal carcinoma histopathology, encompassing its evolution, techniques, and advancements. AI algorithms, notably machine learning and deep learning, have revolutionized image analysis, facilitating accurate diagnosis and prognosis prediction. Furthermore, AI-driven histopathological analysis unveils potential biomarkers and therapeutic targets, heralding personalized treatment approaches. Despite its promise, challenges persist, including data quality, interpretability, and integration. Collaborative efforts among researchers, clinicians, and AI developers are imperative to surmount these hurdles and realize AI's full potential in colorectal carcinoma care. This review underscores AI's transformative impact and implications for future oncology research, clinical practice, and interdisciplinary collaboration.

## Introduction and background

Colorectal carcinoma, also known as colorectal cancer, is a malignant tumor that arises from the colon or rectum. It is one of the most prevalent cancers globally, with significant morbidity and mortality rates. Colorectal carcinoma typically develops from adenomatous polyps and progresses through various stages, posing a substantial health burden worldwide [1]. Histopathology plays a pivotal role in the diagnosis and prognosis of colorectal carcinoma. Through the microscopic examination of tissue samples obtained from biopsies or surgical resections, histopathologists can assess the morphological characteristics of the tumor, including its differentiation, invasion depth, lymphovascular invasion, and tumor budding. These histopathological features provide crucial information for determining the stage of the disease, guiding treatment decisions, and predicting patient outcomes [2].

Artificial intelligence (AI) encompasses diverse technologies that enable computers to perform tasks that typically require human intelligence, such as learning, reasoning, and problem-solving. In recent years, AI has emerged as a powerful tool in healthcare, offering innovative solutions for various clinical applications, including medical imaging interpretation, disease diagnosis, treatment planning, and patient management. AI algorithms can analyze large volumes of medical data, extract meaningful insights, and assist healthcare professionals in making more accurate and timely decisions [3].

This review aims to explore the pioneering role of AI in colorectal carcinoma histopathology. Specifically, we will examine the integration of AI techniques with traditional histopathological methods for diagnosing, prognosis, and managing colorectal cancer. By synthesizing current research findings and technological advancements, this review aims to provide insights into the potential benefits, challenges, and future directions of AI-driven approaches in improving the care of patients with colorectal carcinoma.

## Review

Traditional histopathology in colorectal carcinoma

Histopathological Examination Process

The histopathological examination is a meticulous process involving the microscopic analysis of tissues extracted from biopsies or surgical procedures to diagnose various diseases. Skilled pathologists conduct this examination by meticulously sectioning, staining, and scrutinizing tissue samples under a microscope, identifying cellular changes that signify different conditions [4-6]. This examination is pivotal in diagnosing a spectrum of ailments, encompassing cancer, infectious diseases, inflammatory conditions, autoimmune disorders, as well as organ-specific diseases like endometriosis and uterine fibroids [4,6]. The process begins with preparing tissue sections on glass slides, followed by staining with dyes to enhance visibility, allowing for the meticulous examination of cell structures and groupings to ensure precise diagnoses [4,6]. In the context of cancer, histopathology plays a critical role in diagnosis, staging, and treatment planning by differentiating between benign and malignant tumors and assessing treatment efficacy [6]. Furthermore, advancements in molecular pathology techniques such as fluorescence in-situ hybridization (FISH) and polymerase chain reaction (PCR) have revolutionized cancer management by enabling pathologists to map genetic material in tissues, providing invaluable insights into the molecular mechanisms underlying diseases [6]. Overall, histopathological examination is a cornerstone in understanding disease processes, guiding treatment strategies, and improving patient outcomes. Its meticulous nature and ability to provide insights at the cellular and molecular levels make it indispensable in diagnosing and managing a wide range of medical conditions.

Challenges and Limitations of Traditional Methods

Traditional methods utilized in the diagnosis and treatment of colorectal cancer face a multitude of challenges and limitations despite their pivotal role in healthcare. One significant hurdle is the potential inadequacy of colonoscopy, particularly in the proximal colon, which can impede its effectiveness in detecting neoplasia [7]. Moreover, concerns persist regarding the elevated costs associated with screening strategies, including expenses related to biological therapy for advanced colorectal cancer, which may pose challenges to the cost-effectiveness of colorectal cancer screening programs in the future [7]. The efficacy of traditional methods is further influenced by factors such as compliance with screening protocols and the accuracy of screening tests. However, ongoing issues with low compliance rates are observed in population-based screening programs, highlighting an urgent need for enhanced strategies to bolster participation rates and overall effectiveness [8]. Additionally, uncertainties linger regarding the specificity and reproducibility of various screening tests, such as fecal tests and endoscopic procedures, potentially compromising the reliability and accuracy of traditional colorectal cancer screening methods [8]. These challenges underscore the imperative for continuous advancements and innovations in colorectal cancer screening. Addressing the limitations inherent in traditional approaches through these advancements is essential for improving patient outcomes and augmenting the effectiveness of colorectal cancer detection and treatment programs. By leveraging cutting-edge technologies and implementing novel strategies, healthcare providers can strive towards more robust and efficient colorectal cancer screening initiatives, ultimately benefiting patient care and public health.

Importance of Accurate Diagnosis for Treatment Planning

The importance of accurate diagnosis in treatment planning cannot be overstated, as it serves as a cornerstone in customizing clinical decisions and ensuring optimal health outcomes for patients. Precise diagnosis equips healthcare providers with the necessary information to make informed decisions, accurately identify the underlying causes of symptoms, and formulate effective treatment strategies [9-11]. By reducing diagnostic uncertainty, accurate diagnosis facilitates better recovery outcomes and supports individuals throughout their healing journey by accurately guiding clinical decision-making to address the patient's health concerns [9,10]. Furthermore, accurate diagnosis significantly influences various factors crucial for determining an individual's future health prognosis. These factors include selecting appropriate treatments, assessing treatment responsiveness, evaluating potential treatment-related toxicity, estimating survival rates, improving physical well-being, and considering social factors impacting overall health outcomes [9]. Diagnostic testing, encompassing modalities such as medical imaging (e.g., MRI and bone scans), is a fundamental component of modern medicine, aiding in the precise identification of conditions and guiding the development of tailored treatment plans for enhanced patient care [10]. Timely and accurate diagnosis holds paramount importance in healthcare, as it minimizes errors, empowers patients, and informs comprehensive treatment planning. By delivering personalized and effective care based on a thorough understanding of the patient's condition, accurate diagnosis plays a pivotal role in achieving positive health outcomes and improving overall patient well-being [9,10].

Introduction of AI in histopathology

Overview of AI and Its Applications in Healthcare

AI has evolved remarkably in the healthcare sector, introducing groundbreaking solutions that reshape patient care, diagnosis, treatment, and operational workflows. Its applications span various functions, including diagnosis and treatment recommendations, patient engagement, medication adherence, and administrative tasks [12,13]. By harnessing AI technologies such as machine learning and deep learning, healthcare professionals can sift through vast datasets to provide accurate diagnoses, customize personalized treatment plans, and utilize predictive analytics to improve patient outcomes [14,15]. Importantly, AI in healthcare is not designed to supplant human professionals but to augment their expertise across various domains. From simplifying administrative processes to enhancing clinical documentation and patient monitoring, AI is a valuable ally to healthcare professionals [13]. The potential advantages of integrating AI into healthcare are manifold, encompassing improved patient outcomes, reduced errors, cost savings, and heightened operational efficiency within healthcare facilities [14,15]. The incorporation of AI tools into healthcare operations is expected to yield substantial cost reductions, promote proactive health management, and streamline care delivery processes, thereby ushering in a transformative era in healthcare provision [13,14]. The proliferation of AI in healthcare is propelled by advancements in technology, bolstered data processing capabilities, and the expansion of the AI talent pool. These factors collectively lay the foundation for a paradigm shift in healthcare technology and service delivery, promising enhanced efficiency and efficacy across the healthcare continuum [13]. As AI continues to evolve and integrate further into healthcare systems, its potential to revolutionize patient care and operational workflows remains immense, heralding a new era of innovation and improvement in healthcare services.

Early Attempts to Apply AI in Histopathology

In the early 1970s, the nascent field of AI began making inroads into histopathology, coinciding with its initial medical applications. At this juncture, the primary focus of AI in healthcare was to assist in diagnostic processes, with a particular emphasis on educational support capacities within medical practice. Among the notable endeavors of this period was the development of the 'MYCIN' expert system, which was specifically engineered to aid in diagnosing infectious diseases [16]. This pioneering application of AI represented a significant milestone in integrating artificial intelligence into the field of pathology, heralding the inception of AI-driven approaches in disease diagnosis. The introduction of 'MYCIN' marked the genesis of utilizing AI techniques in pathology, paving the way for substantial advancements in histopathology image analysis and disease detection that are observed today [17,18]. Building upon the foundational principles established by early AI systems, contemporary efforts in histopathology have leveraged sophisticated AI algorithms, including machine learning and deep learning techniques, to analyze histological images with unprecedented precision and efficiency. These advancements have propelled the field of histopathology into a new era characterized by enhanced diagnostic accuracy and the development of novel tools for disease detection and characterization. The early endeavors to apply AI in histopathology during the 1970s laid the groundwork for the transformative advancements witnessed in modern-day histopathology image analysis and disease detection. The pioneering efforts exemplified by systems like 'MYCIN' paved the way for integrating AI into pathology, catalyzing innovation and progress in the field and ultimately contributing to improved patient care and outcomes.

Evolution of AI Techniques for Image Analysis

The evolution of AI techniques for image analysis has catalyzed transformative changes across diverse industries, automating manual tasks, facilitating real-time decision-making, and unlocking scalable insights. In the early stages, image processing relied heavily on conventional methods such as image enhancement, segmentation, and feature extraction. However, with the advent of AI and Machine Learning (ML), image analysis has reached unprecedented levels of sophistication [19-21]. AI techniques, notably machine learning and deep learning, have revolutionized image analysis by significantly enhancing image classification, recognition, and object detection capabilities. These methodologies empower automated sorting, scene comprehension, and semantic segmentation, enabling many applications such as facial recognition, product detection, and autonomous driving [19-21]. Moreover, AI plays a pivotal role in image enhancement by leveraging super-resolution, noise reduction, and color correction to augment image quality for various downstream tasks [20]. The evolution of AI in image analysis has also spurred the development of anomaly detection, visual recommendation systems, content moderation, and image generation capabilities. These advancements find utility in various domains, including defect detection, medical diagnosis, security, and creative image generation, underscoring AI's versatility and profound impact on visual data processing [19-21]. Overall, the evolution of AI techniques for image analysis has significantly expanded the horizons of traditional image processing, empowering industries to optimize operations, reduce costs, explore new revenue streams, and gain competitive advantages through the potent capabilities of AI-driven visual data analysis [19-21].

AI techniques in colorectal carcinoma histopathology

Machine Learning Algorithms for Image Classification

Machine learning algorithms play a pivotal role in image classification, offering a variety of approaches to analyze and categorize images effectively. Supervised learning methods, such as support vector machines (SVMs) and K-nearest neighbors (KNN), leverage labeled training data to classify images into predefined classes with remarkable accuracy [22,23]. SVMs are adept at segregating data into distinct classes and are well-suited for handling high-dimensional data, making them an excellent choice for image classification tasks [23]. Conversely, KNN determines the class of an unlabeled data point based on its closest neighbors, making it a robust option for small to medium-sized datasets [23]. In addition to traditional machine learning algorithms, deep learning models like convolutional neural networks (CNNs) and deep belief networks (DBNs) have demonstrated exceptional performance in image classification tasks. CNNs, in particular, are renowned for their ability to automatically extract features from images through convolutional layers, enabling them to discern intricate patterns and achieve high accuracy in classification tasks [23,24]. On the other hand, DBNs leverage unsupervised learning to pre-train multiple layers before fine-tuning with labeled data, resulting in impressive outcomes in image classification endeavors [23]. Overall, the combination of traditional machine learning algorithms and advanced deep learning models has significantly advanced the field of image classification, allowing for more accurate and efficient analysis of visual data across various domains. These algorithms and techniques drive innovation and progress in image processing and computer vision applications.

Deep Learning Architectures for Feature Extraction

Deep learning architectures for feature extraction play a crucial role across various applications, from image parsing and face recognition to handwriting recognition and medical diagnosis. Among these architectures, CNNs are particularly noteworthy. They are designed to extract and classify image features autonomously, making them highly efficient and well-suited for real-world image analysis tasks [25]. What sets CNNs apart is their seamless integration of feature extraction and classification processes, in contrast to conventional techniques that entail distinct stages for these tasks. This integration contributes to heightened accuracy and performance in various applications, including cancer diagnosis and prognosis evaluation [26,27]. By leveraging the hierarchical structure of CNNs, these networks can automatically learn and extract meaningful features from raw input data, facilitating more precise and effective analysis. Furthermore, the use of ensembles of CNNs has been proposed as a means to enhance feature extraction capabilities and overall performance, surpassing the capabilities of traditional ensemble techniques. This underscores the potential of deep learning approaches in optimizing feature extraction for complex datasets, where capturing intricate patterns and nuances is essential for accurate analysis [25]. Deep learning architectures, particularly CNNs, play a pivotal role in feature extraction across diverse applications, offering superior performance and accuracy in tasks requiring image analysis and pattern recognition. As advancements in deep learning continue to unfold, these architectures hold significant promise for further enhancing our ability to extract and interpret complex features from various data types.

Convolutional Neural Networks for Image Analysis

CNNs represent a specialized form of deep learning algorithm specifically designed for image processing and recognition tasks. Inspired by the hierarchical organization of the visual cortex, these networks have emerged as prominent tools in various computer vision applications, including radiology [28]. Structured to learn spatial hierarchies of features through backpropagation autonomously, CNNs leverage components such as convolution layers, pooling layers, and fully connected layers to accomplish their objectives [28]. A notable advantage of CNNs lies in their ability to extract features hierarchically and progressively, making them highly efficient for image processing tasks. Unlike conventional methods, CNNs eliminate the need for handcrafted feature extraction and can operate without human-expert segmentation of tumors or organs. However, it's important to note that CNNs require substantial data due to their millions of learnable parameters, making them computationally intensive. As a result, graphical processing units (GPUs) are often necessary for model training. Typically, CNNs are structured with convolution and pooling layers followed by fully connected layers. This architecture facilitates the transformation of input data into meaningful outputs via forward propagation [28].

Integration of AI With Traditional Histopathological Techniques

Integrating AI with traditional histopathological techniques marks a significant shift in pathology, promising to enhance diagnostic accuracy and efficiency. By combining AI technologies with established methods like whole slide imaging and manual interpretation, pathologists can leverage AI's computational capabilities to analyze vast datasets and extract insights that may surpass human visual perception [29]. This integration enables the detection of subtle patterns, disease classification, and assessment of biomarkers with enhanced reproducibility and scalability, ultimately refining diagnostic precision and raising the standards of patient care [29]. Furthermore, AI's proficiency in processing complex datasets empowers pathologists to make more informed decisions, paving the way for personalized treatment plans and improved patient outcomes [30,31]. The synergy between AI and traditional histopathological techniques streamlines diagnostic workflows, reducing the time and effort required for analysis and opening up new avenues for research, drug development, and pathology applications [29]. Integrating AI with traditional histopathological methods represents a paradigm shift in pathology, promising to revolutionize diagnostic practices, enhance patient care, and drive innovation in the field. This symbiotic relationship between AI and traditional techniques holds immense potential to shape pathology's future and contribute to healthcare advancements.

Advancements enabled by AI

Improved Accuracy and Efficiency in Diagnosis

The integration of AI has brought about a significant enhancement in the precision and efficiency of diagnosing colorectal cancer. AI technologies, particularly deep-learning-based methods such as Bag of Words and PAHLI, have played a pivotal role in augmenting diagnostic accuracy in the detection of colorectal cancer [32]. These AI systems have demonstrated practicality and yielded favorable outcomes in initial testing, showcasing their ability to autonomously extract intricate features from images or videos, thereby improving speed and specificity compared to conventional methods [32]. Moreover, AI has proven effective in predicting microsatellite instability, a crucial factor for stratifying patients for targeted therapies, thereby underscoring its contribution to enhancing diagnostic accuracy in colorectal cancer [32]. Despite these advancements, there remains a need for more diverse patient data to refine the sensitivity and specificity of neural models for even greater accuracy in diagnosing colorectal cancer [32]. The integration of AI technologies has significantly improved the diagnostic process for colorectal cancer, offering practical and efficient solutions that hold promise for further advancements in accuracy and effectiveness. With continued developments and access to diverse patient data, AI stands poised to continue revolutionizing colorectal cancer diagnosis and improving patient outcomes in the future.

Prediction of Prognosis Based on Histopathological Features

AI tools have significantly advanced the prediction of prognosis based on histopathological features in pathology. By analyzing morphological characteristics from histological images, AI algorithms have shown remarkable capabilities in forecasting patient prognosis and response to specific therapies [31,33]. These tools excel in stratifying patients with varying disease grades, sometimes surpassing the abilities of pathologists [34]. By incorporating factors such as tumor grade, subtype, microenvironment patterns, and genetic profiles, AI algorithms can establish connections between pathology images, survival outcomes, and treatment responses, facilitating a more nuanced approach to precision medicine [31]. Additionally, AI's capacity to predict immunohistochemical marker expression from hematoxylin and eosin (H&E) findings is indispensable for diagnostic purposes, particularly in cases with borderline morphology where traditional methods may falter [31].

Identification of Biomarkers and Therapeutic Targets

Identifying biomarkers and therapeutic targets is critical to advancing precision medicine, particularly within AI-driven platforms like PandaOmics. These platforms harness artificial intelligence and bioinformatics techniques to scrutinize omics and biomedical data, generating novel hypotheses regarding therapeutic targets and biomarkers possessing specific properties [35,36]. The targets and biomarkers pinpointed by these platforms undergo thorough validation via both in vitro and in vivo studies, underscoring their potential clinical significance [35]. As an integral part of Insilico Medicine's Pharma.ai suite, PandaOmics assumes a central role in drug discovery by swiftly identifying molecular targets and biomarkers for various diseases, highlighting the efficacy of AI in expediting the development of precision medicine solutions [35]. Moreover, the utilization of AI in biomarker discovery is anticipated to refine patient stratification, enhance treatment efficacy, and optimize clinical trial outcomes by facilitating more personalized and targeted therapies grounded in specific biomarkers identified through advanced technologies [36].

Enhancing Personalized Treatment Strategies

**Figure 1:**
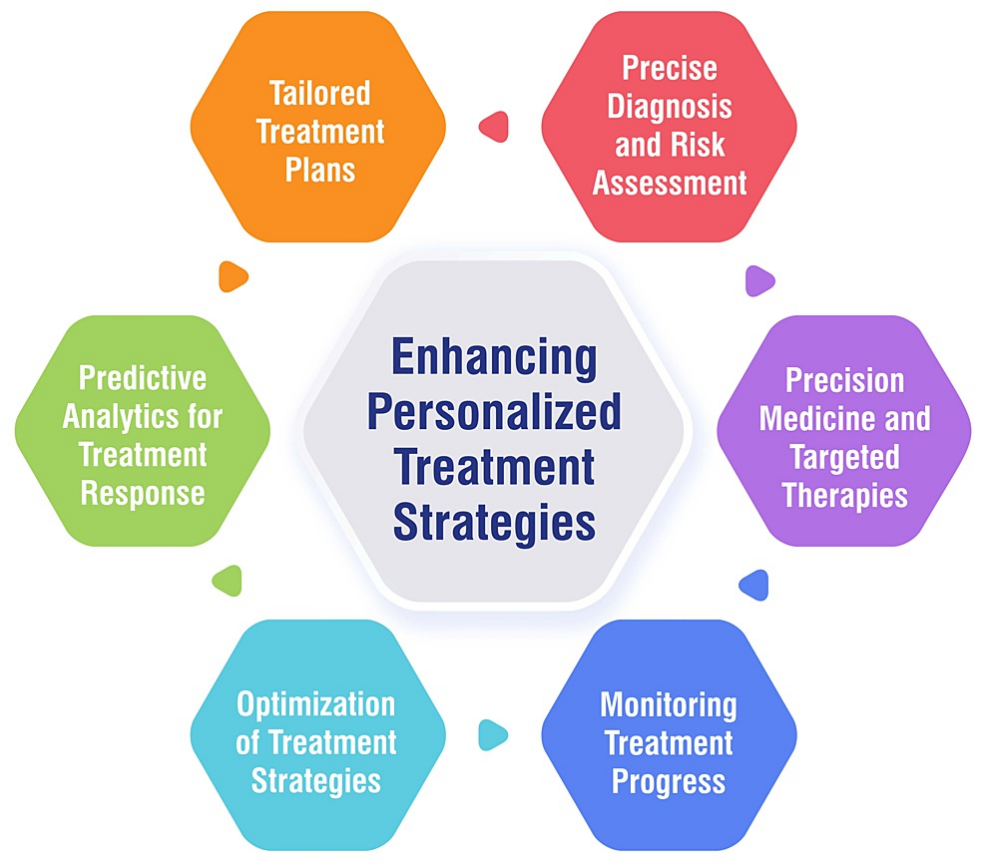
Enhancing personalized treatment strategies Image Credit: Dr. Suhit Naseri

AI is revolutionizing the healthcare sector with its capability to make precise diagnoses and assess risks effectively. By analyzing a wide array of patient data, AI algorithms can uncover disease predispositions, forecast the progression of diseases, and suggest preventive actions. This critical insight enables healthcare professionals to undertake earlier interventions and conduct targeted screenings, significantly improving patient outcomes [37]. Furthermore, AI's role extends to the creation of tailored treatment plans. AI facilitates the customization of treatment plans by delving into patient-specific data, including genetics, biomarkers, comorbidities, and responses to past treatments. This allows healthcare providers to design treatments that are closely aligned with the individual characteristics of each patient, thereby optimizing the therapeutic results [37]. Predictive analytics, another forte of AI, plays a vital role in forecasting treatment responses and outcomes. AI can identify subsets of patients likely to exhibit positive or adverse reactions to specific treatments by analyzing patient data. This predictive capability aids healthcare providers in making more informed decisions, ultimately enhancing patient care [37]. AI also significantly contributes to the optimization of treatment strategies. It considers factors such as patient preferences, resource distribution, cost-effectiveness, and adherence to clinical guidelines to evaluate various treatment options. Consequently, AI can recommend the most suitable course of action, leading to improved patient outcomes and more informed decision-making processes [38]. Monitoring treatment progress is yet another area where AI has made substantial inroads. Utilizing AI-powered monitoring systems, healthcare providers can track the real-time responses of patients to treatments. These systems analyze data from various sources, including wearable devices, electronic health records, and patient-reported outcomes. The insights gained from this analysis are invaluable, enabling timely interventions or adjustments to the treatment plan if necessary [38]. Lastly, AI is at the forefront of advancing precision medicine and targeted therapies. It identifies specific biomarkers, genetic variations, or molecular signatures linked to diseases. This capability enables the development of personalized treatments that are more effective and have reduced side effects, marking a significant step forward in improving patient care [38]. Enhancing personalized treatment strategies are shown in Figure 1.

Challenges and limitations

Data Quality and Quantity for Training AI Models

The significance of data quality and quantity in training AI models cannot be overstated, as these factors are pivotal in ensuring the accuracy, reliability, and efficacy of artificial intelligence systems [39-42]. High-quality data lays the groundwork for AI systems to accurately understand and respond to human inquiries, allowing them to function as designed [39]. While the quantity of data provides the necessary volume for comprehensive training and validation, the quality guarantees that AI models are precise, dependable, and free from bias [40,43,44]. Achieving an equilibrium between data quality and quantity is paramount in developing AI models that yield consistent and trustworthy outcomes [43]. Data quality is characterized by accuracy, reliability, consistency, and completeness. Conversely, data quantity enhances the model's capability to discern patterns and render accurate forecasts. It is critical to ensure that the training data mirrors the diversity of real-world scenarios through meticulous evaluation, curation, and ongoing quality assessments. This approach is essential for AI systems to formulate informed and impartial decisions. Determining the perfect balance between data quality and quantity is crucial for training AI models capable of delivering precise predictions and optimal performance. This underscores the importance of professional practices in data acquisition, cleaning, labeling, and annotation to secure high-quality training datasets [39-42].

Interpretability and Transparency of AI-Generated Results

Interpretability and transparency stand as pivotal elements in the realm of AI-generated outcomes. Interpretability zeroes in on comprehending the intricate workings of an AI algorithm, exploring its parameters, functions, and the interconnections within, to offer an in-depth perspective on the algorithm's operational mechanics [45,46]. Transparency, in contrast, pertains to the level of openness associated with the design, development, and operational phases of AI systems, ensuring that the underpinnings, data inputs, and decision-making frameworks are accessible and comprehensible to stakeholders [45,47]. To achieve a state of interpretability, various methodologies are employed, including using inherently interpretable models, the adoption of transparent learning approaches, and the conduct of empirical validations. These strategies aim to demystify the AI model's functionality, clarifying how it processes and analyzes data [45,46]. Transparency, meanwhile, is indispensable for fostering trust and accountability within AI systems. It is particularly crucial when latent biases could inadvertently influence outcomes, highlighting the necessity for AI systems to be transparent in their decision-making processes [45,47]. At its core, interpretability delves into the AI algorithms' internal machinations. At the same time, transparency ensures an open and comprehensible approach to AI implementations' design and decision-making aspects. Together, these principles are fundamental to the ethical deployment of AI technologies, ensuring they are both understandable and accountable in their operations.

Integration With Existing Healthcare Systems

The seamless integration of AI within existing healthcare frameworks is imperative for unleashing its transformative potential on patient care and clinical outcomes. For AI systems to effectively enhance healthcare services, they must navigate the pathway of regulatory approvals, be harmoniously integrated with Electronic Health Record (EHR) systems, and adhere to rigorous standards to ensure seamless assimilation into current medical practices [12]. This integration process demands significant investments in resources, infrastructure, expertise, and crucial support from clinical stakeholders, encompassing end-users, departments, and entire institutions, to guarantee its enduring efficacy and adaptability [48]. Furthermore, the collaboration across the healthcare ecosystem, involving healthcare entities, AI technologists, and regulatory authorities, is vital in laying down the foundational guidelines and standards that will govern the deployment of AI algorithms within clinical environments [49]. Equally important is the concerted effort to tackle inherent challenges such as ensuring data quality, safeguarding patient privacy, mitigating algorithmic bias, and maintaining an indispensable level of human oversight to foster responsible and efficacious AI applications in healthcare settings [14].

Regulatory and Ethical Considerations

Regulatory and ethical considerations are paramount in deploying AI within healthcare settings, particularly when diagnosing and managing colorectal cancer. These considerations are crucial for safeguarding patient safety, ensuring data privacy, and promoting the responsible application of AI technologies. According to the literature, addressing the legal and ethical challenges associated with AI in healthcare is imperative. These include concerns over privacy, potential biases, discrimination, and the overall safety of patients [50,51]. Moreover, there's a pronounced need for comprehensive regulatory frameworks specifically designed to oversee the use of AI in clinical environments. Such frameworks are essential for maintaining ethical integrity, safeguarding patient rights, and ensuring that AI applications contribute positively to patient care without compromising ethical standards [52,53].

Future directions

Further Refinement of AI Algorithms for Histopathological Analysis

The refinement of AI algorithms for histopathological analysis encompasses several critical aspects, as delineated in the cited sources. Initially, employing more controlled datasets during the early phases of deploying AI/ML algorithms is imperative, setting the stage for subsequent refinements as these algorithms evolve [54]. Furthermore, shifting focus towards gland segmentation models, rather than relying solely on machine learning classifiers, can significantly enhance the accuracy and efficiency of AI models in histopathological analysis [55]. Addressing challenges such as false positives is another essential component of this refinement process. Continuous training and validation of composite AI models are vital strategies for enhancing diagnostic precision and minimizing errors in the histopathological screening of colorectal cancer [55]. Additionally, the expansion of datasets to include larger sample sizes and the execution of multi-site clinical validations are crucial measures to ensure the algorithms' robustness and their generalizability across different histopathological contexts [55].

Integration With Other Diagnostic Modalities for Comprehensive Assessment

The fusion of AI with various diagnostic tools marks a significant advancement in medical diagnostics, especially within radiology. AI algorithms are redefining the landscape of diagnosis by augmenting the precision of image analysis and significantly curtailing the rate of diagnostic inaccuracies [56]. This synergy between AI and diagnostic imaging techniques, including X-ray, CT scans, MRI, and PET scans, equips medical practitioners with a more integrated perspective on patient health, enhancing the accuracy of diagnoses and the customization of treatment protocols [57]. Such integration empowers the handling and analyzing of extensive datasets, unveiling patterns and insights that might elude manual detection. This capability not only elevates diagnostic accuracy but also streamlines the efficiency of the diagnostic process [57].

Adoption of AI in Clinical Practice and Decision-Making

Integrating AI into clinical practice and decision-making represents a rapidly evolving frontier with profound implications for healthcare. The research underscores the potential advantages of AI tools in augmenting clinical decision-making processes, furnishing healthcare professionals with precise, timely, and tailored insights to inform their practice [58,59]. Leveraging machine learning and deep learning technologies, AI systems exhibit promise in forecasting and categorizing diagnoses, proffering recommendations, and enhancing practitioner efficacy across diverse medical domains, including cancer diagnosis and risk assessment [58]. Furthermore, adopting AI in decision-making can elevate healthcare services' caliber, efficiency, and efficacy, ultimately culminating in superior patient outcomes and heightened user satisfaction [58]. As AI technology progresses, it stands poised to revolutionize clinical workflows, standardize decision-making protocols, and alleviate the daily burdens encountered by clinicians by furnishing data-driven, personalized recommendations [59]. The insights gleaned underscore the importance of comprehending AI perception, expectancy, and perceived risk in shaping clinicians' inclination toward utilizing AI-driven decision support systems. This underscores the imperative for AI developers to prioritize elucidating the benefits, ease of use, system potential, and risk perception to facilitate enhanced adoption of AI within healthcare settings [59].

Potential Impact on Patient Outcomes and Healthcare Delivery

The integration of AI into healthcare, particularly in managing colorectal cancer, holds immense promise for positively impacting patient outcomes and the delivery of healthcare services. AI-driven predictive analytics have the potential to significantly enhance the precision, efficiency, and cost-effectiveness of disease diagnosis and clinical laboratory testing, ultimately resulting in superior patient care and outcomes [49]. By harnessing AI algorithms to analyze variables such as population demographics and disease prevalence continuously, healthcare systems can identify individuals at elevated risk of specific conditions, thereby facilitating proactive prevention or treatment strategies [49]. Furthermore, AI technologies such as ML and natural language processing (NLP) can furnish practitioners with real-time, accurate information, streamlining tasks, bolstering efficiency, and alleviating physician stress [49]. In colorectal cancer treatment, AI applications offer personalized, evidence-based clinical treatment strategies that can potentially optimize patient care and outcomes [60]. The development of AI-assisted clinical tools for decision support in managing colorectal cancer, such as predicting future endoscopic surveillance intervals, exemplifies AI's capacity to enhance treatment planning and patient monitoring [61].

## Conclusions

In conclusion, integrating AI into colorectal carcinoma histopathology has undeniably transformed the landscape of cancer diagnosis and management. Through machine learning and deep learning algorithms, AI has demonstrated remarkable accuracy and efficiency in analyzing histopathological images, providing invaluable insights for clinicians. This advancement holds immense potential for improving patient outcomes by enabling more precise diagnosis, prognosis prediction, and personalized treatment planning. However, realizing the full benefits of AI in this domain requires collaborative efforts among researchers, clinicians, and AI developers. Together, they must address challenges such as data standardization, algorithm validation, and ethical considerations to ensure the responsible and effective implementation of AI technologies in clinical practice. Despite these challenges, the future of colorectal carcinoma histopathology appears promising, with AI poised to revolutionize the field and ultimately enhance the care of individuals affected by this prevalent and often devastating disease.

## References

[1] (2024). Colorectal cancer. https://www.who.int/news-room/fact-sheets/detail/colorectal-cancer.

[2] Fleming M, Ravula S, Tatishchev SF, Wang HL (2012). Colorectal carcinoma: pathologic aspects. J Gastrointest Oncol.

[3] Bajwa J, Munir U, Nori A, Williams B (2021). Artificial intelligence in healthcare: transforming the practice of medicine. Future Healthc J.

[4] (2024). What Is Histopathology?. https://www.verywellhealth.com/histopathology-2252152.

[5] Gurina TS, Simms L (2024). Histology, staining. StatPearls.

[6] (2024). Histopathology. https://www.rcpath.org/discover-pathology/news/fact-sheets/histopathology.html.

[7] Quintero E, Hassan C, Senore C, Saito Y (2012). Progress and challenges in colorectal cancer screening. Gastroenterol Res Pract.

[8] Iragorri N, Spackman E (2018). Assessing the value of screening tools: reviewing the challenges and opportunities of cost-effectiveness analysis. Public Health Rev.

[9] (2024). Understanding the Importance of Prompt and Accurate Diagnosis. https://mskradiologypartners.com.au/understanding-prompt-accurate-diagnosis/.

[10] (2024). The Importance of Diagnostic Testing in Your Treatment Plan. Medical Rehabilitation Centers.

[11] (2024). Best Practices Around Providing an Accurate Diagnosis. https://www.mcleanhospital.org/video/diagnostics-best-practices-around-providing-accurate-diagnosis.

[12] Davenport T, Kalakota R (2019). The potential for artificial intelligence in healthcare. Future Healthc J.

[13] Bohr A, Memarzadeh K (2020). The rise of artificial intelligence in healthcare applications. Artif Intell Healthc.

[14] (2024). Artificial Intelligence (AI) in Healthcare & Medical Field. https://www.foreseemed.com/artificial-intelligence-in-healthcare.

[15] Sharma M, Savage C, Nair M, Larsson I, Svedberg P, Nygren JM (2022). Artificial intelligence applications in health care practice: scoping review. J Med Internet Res.

[16] Moxley-Wyles B, Colling R, Verrill C (2020). Artificial intelligence in pathology: an overview. Diagn Histopathol.

[17] Abdelsamea MM, Zidan U, Senousy Z, Gaber MM, Rakha E, Ilyas M (2022). A survey on artificial intelligence in histopathology image analysis. WIREs Data Min Knowl Discov.

[18] Scavuzu A (2023). The role of artificial intelligence in histopathology: a comprehensive overview. J Cytol.

[19] (2024). What is AI Image Processing? Your Quick Guide. https://www.klippa.com/en/blog/information/ai-image-processing/.

[20] K L (2024). The Power of AI in Image Processing: A Comprehensive Guide. Vegavid Technology.

[21] (2024). Artificial Intelligence and Machine Learning based Image Processing. https://www.design-reuse.com/articles/53213/artificial-intelligence-and-machine-learning-based-image-processing.html.

[22] Boesch G (2024). A Complete Guide to Image Classification in 2024. in.

[23] (2024). Mastering Image Classification Techniques: Boosting Accuracy and Efficiency. https://www.linkedin.com/pulse/mastering-image-classification-techniques-boosting-accuracy-kanjee/.

[24] (2023). Image Recognition: Definition, Algorithms & Uses. https://www.v7labs.com/blog/image-recognition-guide.

[25] Shaheen F, Verma B (2016). An ensemble of deep learning architectures for automatic feature extraction. 2016 IEEE Symposium Series on Computational Intelligence (SSCI).

[26] Weimer D, Scholz-Reiter B, Shpitalni M (2016). Design of deep convolutional neural network architectures for automated feature extraction in industrial inspection. CIRP Ann.

[27] Çayir A, Yenidoğan I, Dağ H (2018). Feature extraction based on deep learning for some traditional machine learning methods. UBMK.

[28] Yamashita R, Nishio M, Do RK, Togashi K (2018). Convolutional neural networks: an overview and application in radiology. Insights Imaging.

[29] Baxi V, Edwards R, Montalto M, Saha S (2022). Digital pathology and artificial intelligence in translational medicine and clinical practice. Mod Pathol.

[30] Cui M, Zhang DY (2021). Artificial intelligence and computational pathology. Lab Invest.

[31] Rakha EA, Toss M, Shiino S, Gamble P, Jaroensri R, Mermel CH, Chen PC (2021). Current and future applications of artificial intelligence in pathology: a clinical perspective. J Clin Pathol.

[32] Uchikov P, Khalid U, Kraev K (2024). Artificial intelligence in the diagnosis of colorectal cancer: a literature review. Diagnostics (Basel).

[33] Shafi S, Parwani AV (2023). Artificial intelligence in diagnostic pathology. Diagn Pathol.

[34] Tolkach Y, Wolgast LM, Damanakis A (2023). Artificial intelligence for tumour tissue detection and histological regression grading in oesophageal adenocarcinomas: a retrospective algorithm development and validation study. Lancet Glob Health.

[35] Kamya P, Ozerov IV, Pun FW (2024). PandaOmics: an AI-driven platform for therapeutic target and biomarker discovery. J Chem Inf Model.

[36] Beaney A (2023). Precision medicine: Promising future as AI, biomarkers and technology bolster the field. Clinical Trials Arena.

[37] (2024). AI in Medicine: Transforming Patient Treatment and Care. https://www.thoughtful.ai/blog/ai-in-medicine-transforming-patient-treatment-and-care.

[38] Yelne S, Chaudhary M, Dod K, Sayyad A, Sharma R (2023). Harnessing the power of AI: a comprehensive review of its impact and challenges in nursing science and healthcare. Cureus.

[39] (2024). Prioritizing Quality Over Quantity: A Critical Imperative in AI Training. https://www.linkedin.com/pulse/prioritizing-quality-over-quantity-critical-ai-anastasiia-pereverzeva/.

[40] PhD EG: Data Quality vs (2024). Data Quality vs. Data Quantity: The Crucial Balance for Artificial Intelligence. https://python.plainenglish.io/data-quality-vs-data-quantity-the-crucial-balance-for-artificial-intelligence-faed8b0eaea4.

[41] (2024). Explaining Data Quality for Machine Learning. https://www.ayadata.ai/blog-posts/importance-of-data-quality-for-machine-learning/.

[42] (2024). Finding the balance between Data Quality and Data Quantity for an Accurate AI Model. https://dataloop.ai/blog/finding-the-balance-between-data-quality-and-data-quantity-for-an-accurate-ai-model/.

[43] Benishu I (2024). Finding the balance between Data Quality and Data Quantity for an Accurate AI Model. https://dataloop.ai/blog/finding-the-balance-between-data-quality-and-data-quantity-for-an-accurate-ai-model/.

[44] (2024). Data Quality and Quantity for Machine Learning. https://www.monolithai.com/blog/data-quality-and-quantity-for-machine-learning.

[45] (2024). What is AI transparency? A comprehensive guide. https://www.zendesk.com/in/blog/ai-transparency/.

[46] (2024). Explainability vs Interpretability: the challenge of transparent artificial intelligence. https://www.xcally.com/news/explainability-vs-interpretability-the-challenge-of-transparent-artificial-intelligence/.

[47] (2024). Transparency, Explainability, and Interpretability of AI. https://ediscoverytoday.com/2023/12/12/transparency-explainability-and-interpretability-of-ai-artificial-intelligence-best-practices/.

[48] Kwong JC, Nickel GC, Wang SC, Kvedar JC (2024). Integrating artificial intelligence into healthcare systems: more than just the algorithm. NPJ Digit Med.

[49] Alowais SA, Alghamdi SS, Alsuhebany N (2023). Revolutionizing healthcare: the role of artificial intelligence in clinical practice. BMC Med Educ.

[50] Naik N, Hameed BM, Shetty DK (2022). Legal and ethical consideration in artificial intelligence in healthcare: who takes responsibility?. Front Surg.

[51] Yadav A, Kumar A (2023). Artificial intelligence in rectal cancer: what is the future?. Artif Intell Cancer.

[52] Rompianesi G, Pegoraro F, Ceresa CD, Montalti R, Troisi RI (2022). Artificial intelligence in the diagnosis and management of colorectal cancer liver metastases. World J Gastroenterol.

[53] Shreve JT, Khanani SA, Haddad TC (2022). Artificial intelligence in oncology: current capabilities, future opportunities, and ethical considerations. Am Soc Clin Oncol Educ Book.

[54] Rashidi HH, Tran NK, Betts EV, Howell LP, Green R (2019). Artificial intelligence and machine learning in pathology: the present landscape of supervised methods. Acad Pathol.

[55] Ho C, Zhao Z, Chen XF (2022). A promising deep learning-assistive algorithm for histopathological screening of colorectal cancer. Sci Rep.

[56] Najjar R (2023). Redefining radiology: a review of artificial intelligence integration in medical imaging. Diagnostics (Basel).

[57] Milan L (2023). Integrated Diagnostics and Theranostics of Thyroid Diseases.

[58] Khosravi M, Zare Z, Mojtabaeian SM, Izadi R (2024). Artificial intelligence and decision-making in healthcare: a thematic analysis of a systematic review of reviews. Health Serv Res Manag Epidemiol.

[59] Choudhury A (2022). Factors influencing clinicians' willingness to use an AI-based clinical decision support system. Front Digit Health.

[60] Aikemu B, Xue P, Hong H (2020). Artificial intelligence in decision-making for colorectal cancer treatment strategy: an observational study of implementing Watson for oncology in a 250-case cohort. Front Oncol.

[61] Wei MY, Zhang J, Schmidt R, Miller AS, Yeung JM (2023). Artificial intelligence (AI) in the management of colorectal cancer: on the horizon?. ANZ J Surg.

